# Neuroprotective Effects of *Bifidobacterium animalis subsp. lactis* NJ241 in a Mouse Model of Parkinson’s Disease: Implications for Gut Microbiota and PGC-1α

**DOI:** 10.1007/s12035-024-04038-2

**Published:** 2024-02-26

**Authors:** Yuxuan Dong, Yueyan Qi, Jinhu Chen, Siyuan Han, Wenjing Su, Xin Ma, Yang Yu, Yanqin Wang

**Affiliations:** 1 Hebei Research Center of the Basic Discipline of Cell Biology, Hebei Collaborative Innovation Center for Eco-Environment, Hebei Key Laboratory of Physiology, College of Life Sciences, Hebei Normal University, Shijiazhuang, 050024 China; 2 Department of Endocrinology, Hebei General Hospital, Shijiazhuang, 050051 China; 3 Thankcome Biotechnology (Su Zhou) Co., Suzhou, China

**Keywords:** *Bifidobacterium animalis subsp. lactis* NJ241, Parkinson’s disease, GLP-1, Gut microbiota, Neuroinflammation

## Abstract

Intestinal dysbiosis plays a critical role in the pathogenesis of Parkinson’s disease (PD), and probiotics have emerged as potential modulators of central nervous system function through the microbiota-gut-brain axis. This study aimed to elucidate the anti-inflammatory effects and underlying mechanisms of the probiotic strain *Bifidobacterium animalis subsp. lactis* NJ241 (NJ241) in a mouse model of PD induced by 1-methyl-4-phenyl-1,2,3,6-tetrahydropyridine (MPTP). The impact of NJ241 was comprehensively assessed in PD mice through behavioral tests, immunofluorescence, Western blotting, enzyme-linked immunosorbent assay (ELISA), 16S rRNA sequencing, and short-chain fatty acid (SCFA) detection. NJ241 exhibited notable efficacy in mitigating MPTP-induced weight loss, gastrointestinal dysfunction, and behavioral deficits in mice. Furthermore, it demonstrated protected against MPTP-induced dopaminergic neuron death and inhibited the activation of glial cells in the substantia nigra (SN). NJ241 demonstrated the ability to normalized dysbiosis in the intestinal microbiota and elevate SCFA levels in PD mice. Additionally, NJ241 reversed MPTP-induced reductions in colonic GLP-1 levels and the expression of GLP-1R and PGC-1α in the SN. Notably, GLP-1R antagonists partially reversed the inhibitory effects of NJ241 on the activation of glial cells in the SN. In summary, NJ241 exerts a neuroprotective effect against MPTP-induced neuroinflammation by enhancing intestinal GLP-1 levels and activating nigral PGC-1α signaling. These findings provide a rationale for the exploration and development of probiotic-based therapeutic strategies for PD.

## Introduction

Parkinson’s disease (PD) is the second most prevalent neurodegenerative disorder, with an estimated nearly 5 million individuals projected to be affected by PD in China alone by 2030 due to the aging population [1]. The escalating burden of PD on society is a cause for concern. The fundamental pathological features of PD involved the degeneration and death of dopaminergic neurons in the substantia nigra pars compacta (SNc), and the formation of Lewy bodies primarily composed of α-synuclein(α-syn). These abnormalities disrupt the nigrostriatal system, giving rise to motor symptoms such as muscle rigidity, resting tremor, bradykinesia, and gait abnormalities. Notably, contemporary research indicates that PD patients exhibit non-motor symptoms even before the onset of motor dysfunction, encompassing gastrointestinal issues, hyposmia, depression, sleep disorders, and others, profoundly impacting the quality of life. The precise etiology of PD remains elusive, potentially involving genetics, environmental factors, oxidative stress, neuroinflammation, and gut dysbiosis. Braak et al. [2] postulated that α-syn deposition in the intestines might retrogradely transfer to the central nervous system, a notion substantiated by studies demonstrating the transmission of different α-syn variants from the intestine to the brain [3]. Recent population-based studies have suggested a potential association between inflammatory bowel disease and an increased risk of PD. Additionally, the PD-associated pathogenic gene LRRK2 has been linked to Crohn’s disease [4]. Cumulative evidence points toward an intricate interconnection between PD and the intestine, constituting an intestinal-brain axis.

The gut microbiota (GM), often referred to as the “second brain,” harbors 10 times more cells and 150 times more genes than the human genome [5]. Probiotics, defined as live microorganisms that confer health benefits in adequate amounts, have been implicated in various health-promoting effects, including anti-aging, anti-cancer, and cholesterol-lowering properties. Intriguingly, certain probiotics demonstrate modulatory effects on neurological disorders such as Alzheimer’s, PD, and Huntington’s disease [6–11]. *Bifidobacterium*, a crucial probiotic for gut health, maintains stable levels in adults, but these levels decline with aging. *Bifidobacterium* promotes the production of SCFAs, particularly butyrate [12, 13]. SCFAs play a beneficial role in PD by stimulating GLP-1 secretion, ameliorating PD-associated mouse behaviors, preventing DA neuron death, and restoring reduced tight junction protein levels induced by MPTP toxicity [14].

GLP-1, an intestinally derived hormone, binds to its receptor GLP-1R to exert its effects. GLP-1R detectable in SN and its activation demonstrate improvement in behavioral impairment, inhibition of neuroinflammation, and protection of dopaminergic neurons in PD mice [15, 16]. An engineered GLP-1-secreting probiotic strain has been shown to reduce gut *Enterobacteriaceae*, elevate *Lactobacillus* and *Akkermansia*, ameliorate MPTP-induced movement defects, increase tyrosine hydroxylase (TH) neurons, and suppress microglial and astrocyte activation [17].

PGC-1α, a crucial transcriptional coactivator involved in the regulation of cellular metabolism and energy homeostasis, is predominantly expressed in tissues such as the liver, muscle, heart, and brain. Its interaction with various transcription factors modulates inflammatory responses, and decreased expression and gene mutations contribute to the pathogenesis of numerous inflammation-driven diseases [18]. Our laboratory demonstrated that the GLP-1R agonist liraglutide exerts neuroprotection in a PD mouse model by suppressing nigral inflammation and enhancing mitochondrial health via GLP-1R/PGC-1α signaling [19].

Therefore, we hypothesize that probiotics may enhance intestinal GLP-1 secretion, influencing the GLP-1R/PGC-1α signaling pathway in the central nervous system to protect nigral dopaminergic neurons. To investigate whether *Bifidobacterium animalis subsp. lactis* NJ241 (abbreviated as NJ241) increases intestinal GLP-1 expression and has a preventive effect on the occurrence of MPTP-induced PD, and whether this effect is mediated through the GLP-1R/PGC-1α signaling pathway, we intervened with NJ241 in the classic MPTP-induced PD mouse model. Our findings revealed that NJ241 elevated colonic GLP-1 levels, suppressed neuroinflammation, regulated intestinal microbiota and the levels of SCFA, and protected nigral dopaminergic neurons. Notably, the application of a GLP-1R inhibitor attenuated the effects of NJ241.

## Materials and Methods

### Animals and Drug Treatment

Male C57BL/6 mice (20 ± 2 g) were obtained from Liaoning Changsheng Biotechnology and were accommodated in an environment subjected to a 12-h light/dark cycle at a temperature of 25 °C. All procedures involving animals received approval from the Ethics Committee of Hebei Normal University (2023LLSC008) and were conducted in accordance with the guidelines outlined in the Declaration of Helsinki. Vigilant efforts were undertaken to minimize both the distress experienced by the animals and the overall number involved in the study.

**Fig. 1:**
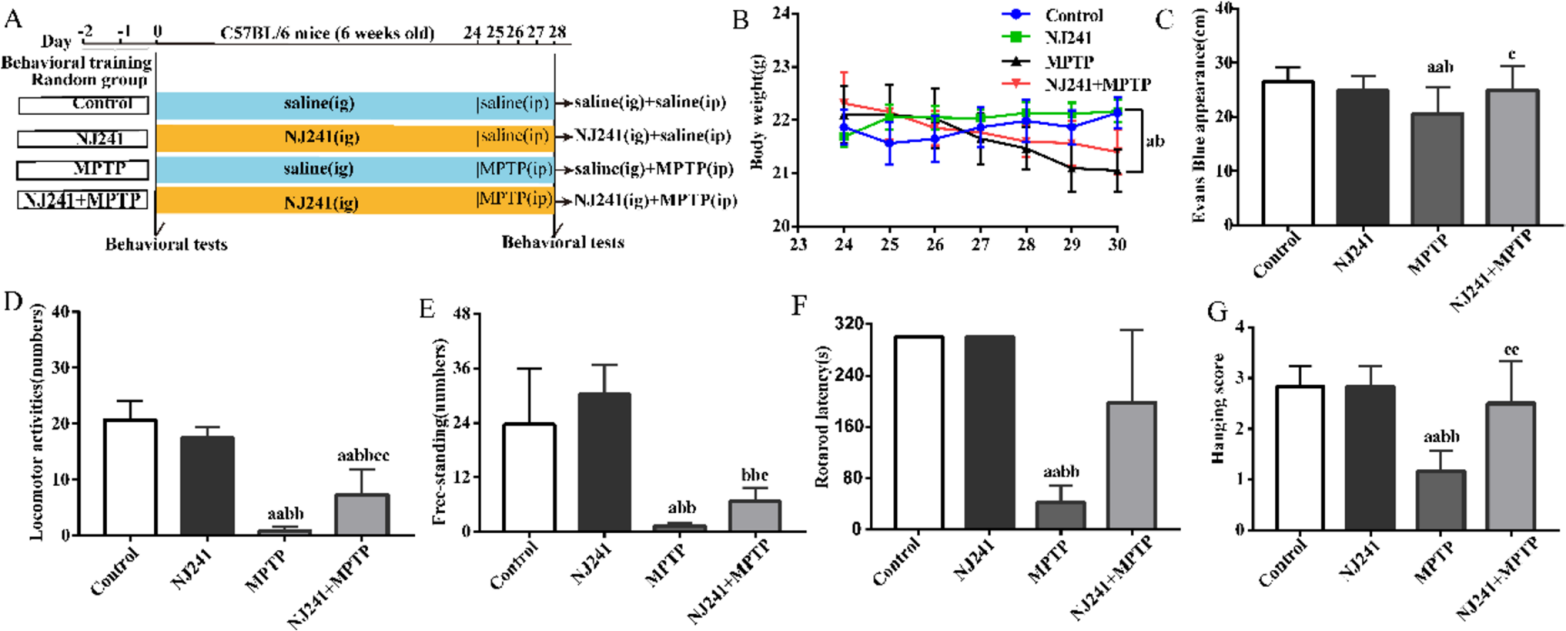
NJ241 mitigates MPTP-induced weight loss, gastrointestinal dysfunction, and behavioral deficits. **A** Experimental timeline. **B** Mouse body weights throughout the study. **C** Small intestinal transit distance of Evans blue dye. Behavioral assessments post-MPTP injection: **D** locomotor activity, **E** rearing, **F** rotarod latency, and **G** wire hanging score. Data are mean ± SEM, *n* = 10/group. a *P* < 0.05, aa *P* < 0.01 vs. control; b *P* < 0.05, bb *P* < 0.01 vs. NJ241; c *P* < 0.05, cc *P* < 0.01 vs. MPTP

Following a 2-day acclimation and training period, the mice were randomly divided into four groups: control, NJ241, MPTP, and NJ241 + MPTP. The control and NJ241 groups were administered saline or NJ241 (1 × 10^9^ CFU/0.2 ml in saline), respectively, through daily oral gavage over a span of 28 days. The MPTP group received saline via gavage and intraperitoneal MPTP (30 mg/kg/0.2 ml saline) injections on days 24–28. The NJ241 + MPTP group underwent NJ241 gavage and MPTP injection following the same schedule. On day 29, behavioral analyses were conducted prior to euthanasia (Fig. 1A).

In the second experiment, mice were randomly assigned to four groups (*n* = 10/group): control, MPTP, NJ241 + MPTP, and NJ241 + MPTP + Exendin (9–39). Exendin (9–39) was administered intraperitoneally at a dosage of 84 μg/kg, 30 min before NJ241 gavage. The administration of MPTP and NJ241 adhered to the same protocols as employed in the initial experiment.

### Reagents

*Bifidobacterium animalis subsp. lactis* NJ241 (CGMCC NO.20816) was obtained from Thankcome Biotechnology Co., Ltd (Suzhou, China). MPTP was purchased from MedChemExpress (Shanghai, China). The antibodies utilized in this study were obtained as follows: rabbit anti-TH, anti-GLP-1R, anti-GFAP, and mouse anti-GAPDH polyclonal antibodies were acquired from Proteintec (Wuhan, China); rabbit anti-Iba-1, anti-PGC-1α, and anti-GLP-1R monoclonal antibodies were sourced from Abcam (Shanghai, China); rabbit anti-ZO-1 polyclonal antibody was obtained from Servicebio (Wuhan, China); rabbit anti-occludin polyclonal antibody was acquired from Wanleibio (Shenyang, China); and rabbit anti-GLP-1 polyclonal antibody, Alexa Fluor 594-conjugated goat anti-rabbit IgG, HRP-conjugated goat anti-mouse IgG, and HRP-conjugated goat anti-rabbit IgG were obtained from Abways Technology (Shanghai, China).

### Behavioral Test

#### Evaluation of Locomotor Activity and Rearing Behavior

In an open-field apparatus, mice were individually situated, and the quantification of horizontal movements (representative of locomotor activity) and vertical movements (indicative of rearing behavior) was conducted over a duration of 5 min in a subdued and darkened environment.

#### Rotarod Test

Prior to the commencement of the experiment, two training sessions were executed, each lasting 120 s and set at a rotation speed of 30 rpm. On the designated test day, the rotarod apparatus was set to a speed of 30 rpm, and the mice were placed on the rotarod. The latency to the first fall within a 5 min interval was recorded.

#### Wire Hanging Test for Motor Coordination Assessment

To assess motor coordination in mice, a hanging test was conducted following the procedure outlined by Hu et al.[20]. A horizontal wire 2 mm in diameter was positioned 50–60 cm above a padded surface. Each mouse was situated on the wire, initially gripping it with its forepaws. The scoring system consisted of assigning 3 points if the wire was grasped by both hind paws and forepaws, 2 points for grasping with one hind paw and both forepaws, 1 point for grasping solely with both forepaws, and 0 point if the mouse lost its grip and fell off.

### Measurement of Weight and Gastrointestinal Motility

Twenty minutes prior to tissue collection, 0.2 ml of a 0.5% Evans blue solution was administered via oral gavage. Following perfusion and fixation, the entire gastrointestinal tract was meticulously excised. The distance covered by the Evans blue solution from the pylorus to the furthest point was measured to valuate gastrointestinal motility.

### Immunofluorescence Analysis

Following transcardial perfusion and fixation with 4% paraformaldehyde, brains and colons were collected, and a post-fixation period of 6 h was employed. Tissues were cryoprotected in 25% sucrose until saturation. Utilizing a microtome, coronal sections of midbrain, containing SN, were obtained with a thickness of 30 μm. Colon tissues were sectioned at 10-μm intervals. The sections underwent a series of procedures, including washing in 0.01 M PBS (3 × 5 min), permeabilization for 15 min, and subsequent washing. Following these preparatory steps, the sections were subjected to an overnight incubation with primary antibodies at room temperature with gentle shaking. The primary antibodies used included rabbit anti-TH (1:2000), rabbit anti-GFAP (1:8000), rabbit anti-Iba-1 (1:4000), rabbit anti-GLP-1R (1:200), rabbit anti-ZO-1 (1:1000), and rabbit anti-Occludin (1:200). After PBS washes (3 × 5 min), the sections underwent a 2-h incubation in the dark with AlexaFluor 594-conjugated goat anti-rabbit IgG secondary antibody (1:400). Following a final series of PBS washes (3 × 5 min), the sections were air dried, mounted with fluorescent medium, and imaged using an Olympus BX51 microscope (GFAP imaging at matched exposures).

### Immunoblotting Analysis

SN and colon tissues that were not fixed underwent rapid freezing in liquid nitrogen and were then stored at – 80 °C. For protein extraction, tissues underwent homogenization in lysis buffer containing RIPA, PMSF, and phosphatase inhibitors (100:1:1 per 10 mg tissue). Homogenates were centrifuged at 12,000 rpm for 15 min at 4 °C, and the resulting supernatants were collected. Protein concentrations were determined using BCA assay, with 20 μg of protein samples prepared in 10 μl volumes.

Proteins were separated via SDS-PAGE, employing 4% concentrated gel and a 10% or 12% separation gels based on the molecular weight of the target protein. Following electrophoretic transfer to PVDF membranes, the membranes were blocked with 5% nonfat milk for 3 h at room temperature, and then incubated overnight at 4 °C with the designated primary antibodies: rabbit anti-TH polyclonal antibody (1:2000), rabbit anti-GFAP polyclonal antibody (1:5000), rabbit anti-ZO-1 polyclonal antibody (1:1000), rabbit anti-Occludin polyclonal antibody (1:1000), or mouse anti-GAPDH polyclonal antibody (1:5000) as needed. After TBST washes (3 × 5 min), HRP-conjugated secondary antibodies were applied at a 1:5000 dilution for 2 h at room temperature. Protein bands were visualized using chemiluminescence (ChemiDocTM, BioRad) and quantified utilizing ImageJ software, normalized to the GAPDH loading control. All experiments were performed in triplicate for each target protein in every mouse.

### Gut Microbiome Analysis

Mouse fecal pellets were systematically collected, promptly flash frozen in liquid nitrogen, and then stored at – 80 °C until processing. Total DNA extraction was executed utilizing the FastDNA® Spin Kit for Soil (Omega Bio-tek). The amplification of the V3–V4 region of bacterial 16S rRNA was achieved through PCR, employing 338F and 806R primers. Amplicons underwent sequencing on an Illumina MiSeq PE300 platform, facilitated by Shanghai Majorbio Bio-pharm Technology Co. The obtained reads were subjected to clustering into operational taxonomic units (OTUs) at 97% similarity, with the removal of chimeras conducted using UPARSE v7.1 [21]. Taxonomic classification was ensued through RDP Classifier v2.2, aligned against the Silva 16S rRNA database (v138) [22]. The assessment of α-diversity involved the application of Chao1 and Simpson indices, while β-diversity was evaluated through principal coordinate analysis (PCoA) of weighted UniFrac distances. The community structure at the phylum level was characterized by relative abundance. Genus-level differences between groups were discerned through a heatmap analysis.

### Short Chain Fatty Acid (SCFA) Analysis

Sample preparation following the Xue method [23] involved homogenizing samples for 1 min with 500 μl of water and 100 mg of glass beads. The homogenates were then centrifuged at 4 ℃ for 10 min at 12,000 rpm. A 200-μl aliquot of the supernatant was extracted using 100 μl of 15% phosphoric acid, 20 μl of a 375 μg/ml 4-methylvaleric acid solution as IS, and 280 μl of ether. Following vortexing for 1 min, the samples underwent another centrifugation at 12,000 rpm for 10 min at 4 ℃, and the resulting supernatant was transferred into a vial for subsequent GC–MS analysis.

GC analysis was conducted using a Trace 1310 gas chromatograph (Thermo Fisher Scientific, USA). equipped with an Agilent HP-INNOWAX capillary column (30 m × 0.25 mm ID × 0.25 μm). Helium serves as the carrier gas at a flow rate of 1 ml/min. The injection, in split mode at 10:1, involved a 1 μl injection volume with injector temperature of 250 ℃. The column temperature program initiated at 90 ℃, ramping up to 120 ℃ at 10 ℃/min, and then to 150 ℃ at 5 ℃/min, and finally to 250 ℃ at 25 ℃/min, maintained for 2 min [24, 25].

Metabolite detection employed an ISQ LT mass spectrometer (Thermo Fisher Scientific, USA) in electron impact ionization mode. Single ion monitoring (SIM) mode was applied with an electron energy of 70 eV [24, 25]. The temperatures of the ion source and MS transfer line were set at 300 °C and 250 °C, respectively.

### Statistical Analysis

The quantification of TH and Iba-1 positive cells in the SN was conducted bilaterally utilizing Photoshop. GFAP immunoreactivity was assessed as the average optical density through the application of Image Pro Plus. Data were analyzed in SPSS 21.0 using one-way ANOVA and are presented as mean ± SEM. Multiple comparisons among experimental groups were performed, and *P* < 0.05 was considered statistically significant.

## Results

### NJ241 Mitigates MPTP-induced Weight Loss, Gastrointestinal Dysmotility, and Behavioral Disorders

The experimental timeline is depicted in Fig. 1A. Throughout the study duration, mouse weights in the control and NJ241 groups exhibited stability. In contrast, both the MPTP and NJ241 + MPTP groups experienced weight loss following MPTP administration until the end of the study (*P* = 0.031, *P* = 0.034).

Baseline behavioral testing, conducted prior to MPTP injection, revealed no discernible differences between groups in locomotor activity, rearing, rotarod performance, or wire hanging ability. Following MPTP administration, the NJ241 group did not deviate significantly from the control group in any measure. However, MPTP-induced mice exhibited substantial reductions in locomotor activity, rearing, rotarod latency, and wire hanging scores in comparison to both the control group (*P* < 0.001, *P* < 0.001, *P* = 0.04, and *P* = 0.001, respectively) and the NJ241 group (*P* < 0.001, *P* < 0.001, *P* = 0.005, and *P* = 0.003, respectively). The intervention with NJ241 resulted in increased locomotor activity, rearing, and wire hanging ability compared to MPTP mice (*P* = 0.001, *P* = 0.045, and *P* = 0.028, respectively), although rotarod latency remained unaltered (*P* > 0.05).

Gastrointestinal motility was evaluated through the oral gavage of Evans blue dye, and the measurement of dye migration distance in the small intestine was conducted after 20 min. In comparison to controls, intestinal transport distance was notably diminished in MPTP mice (Fig. 1C, *P* = 0.003) and significantly lower than the NJ241 group (*P* = 0.018). However, NJ241 + MPTP mice exhibited increased dye migration versus MPTP alone (Fig. 1C, *P* = 0.024).

### NJ241 Alleviates MPTP-induced Damage to Dopaminergic Neurons

**Fig. 2:**
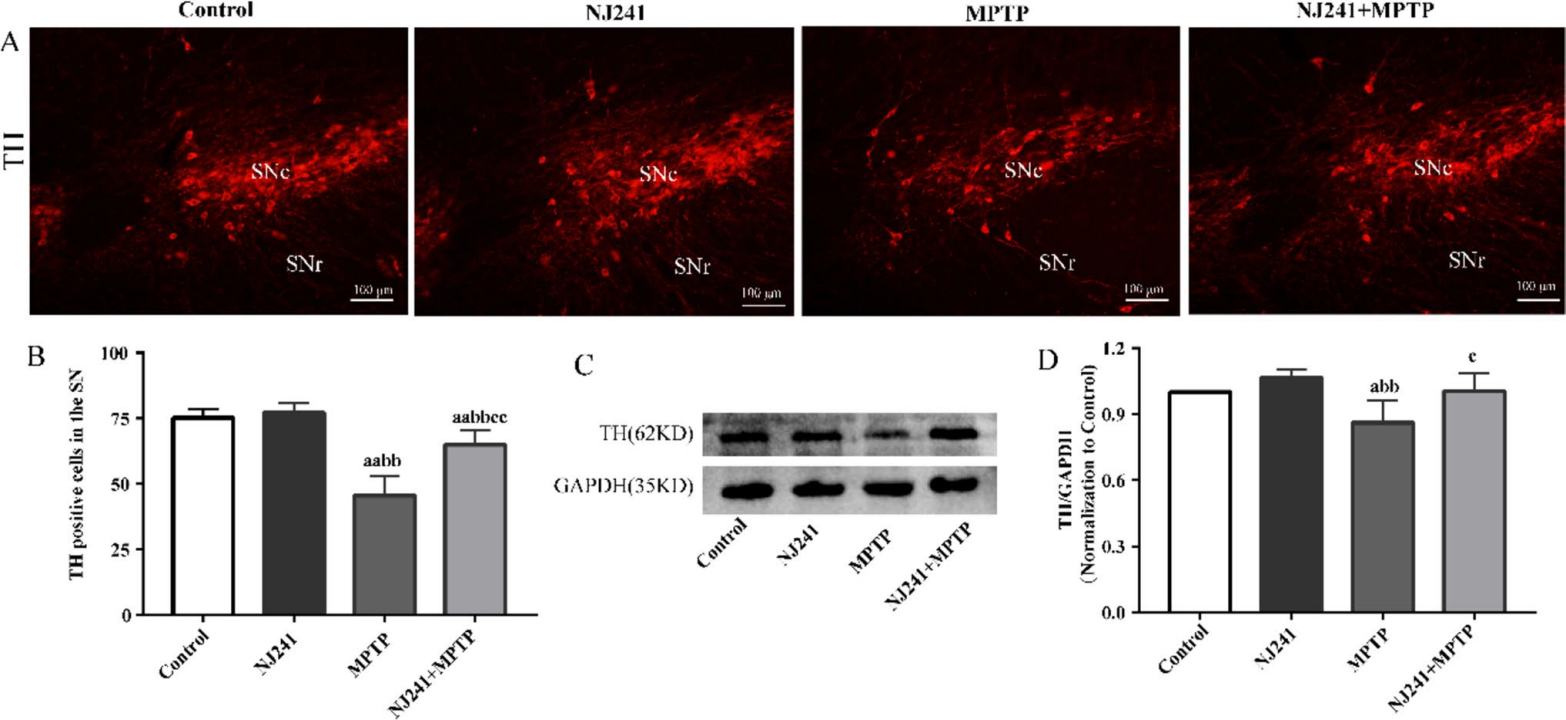
NJ241 protects dopaminergic neurons from MPTP-induced damage. **A** Representative images of TH immunofluorescence in the SNc. **B** Quantification of TH-positive cells (*n* = 6 mice/group). **C** Representative TH and GAPDH immunoblots. **D** Densitometric analysis of TH expression (*n* = 3 mice/group). Data are mean ± SEM. Scale bar = 100 μm. SNc, substantia nigra pars compacta; SNr, substantia nigra pars reticulata. a *P* < 0.05, aa *P* < 0.01 vs. control; bb *P* < 0.01 vs. NJ241; c* P* < 0.05 vs. MPTP

As the rate-limiting enzyme in dopamine synthesis, TH immunoreactivity serves as an indicator of dopaminergic neuron survival. The impact of NJ241 on TH expression in the SN was evaluated through immunofluorescence and immunoblotting. TH-positive cell counts and TH protein levels exhibited no significant differences between the NJ241 and control group. Contrastingly, MPTP-induced mice demonstrated pronounced reductions in SN TH-positive cells compared to controls (Fig. 2D, *P* < 0.001). TH protein expression was also diminished in MPTP mice relative to controls (Fig. 2F, *P* = 0.037) and NJ241-treated mice (Fig. 2F, *P* = 0.006). NJ241 intervention resulted in increased TH-positive cells (Fig. 2D, *P* < 0.001) and elevated TH protein expression (Fig. 2F, *P* = 0.031) compared to MPTP-induced mice.

### NJ241 Inhibits MPTP-induced Glial Activation in the SN

**Fig. 3:**
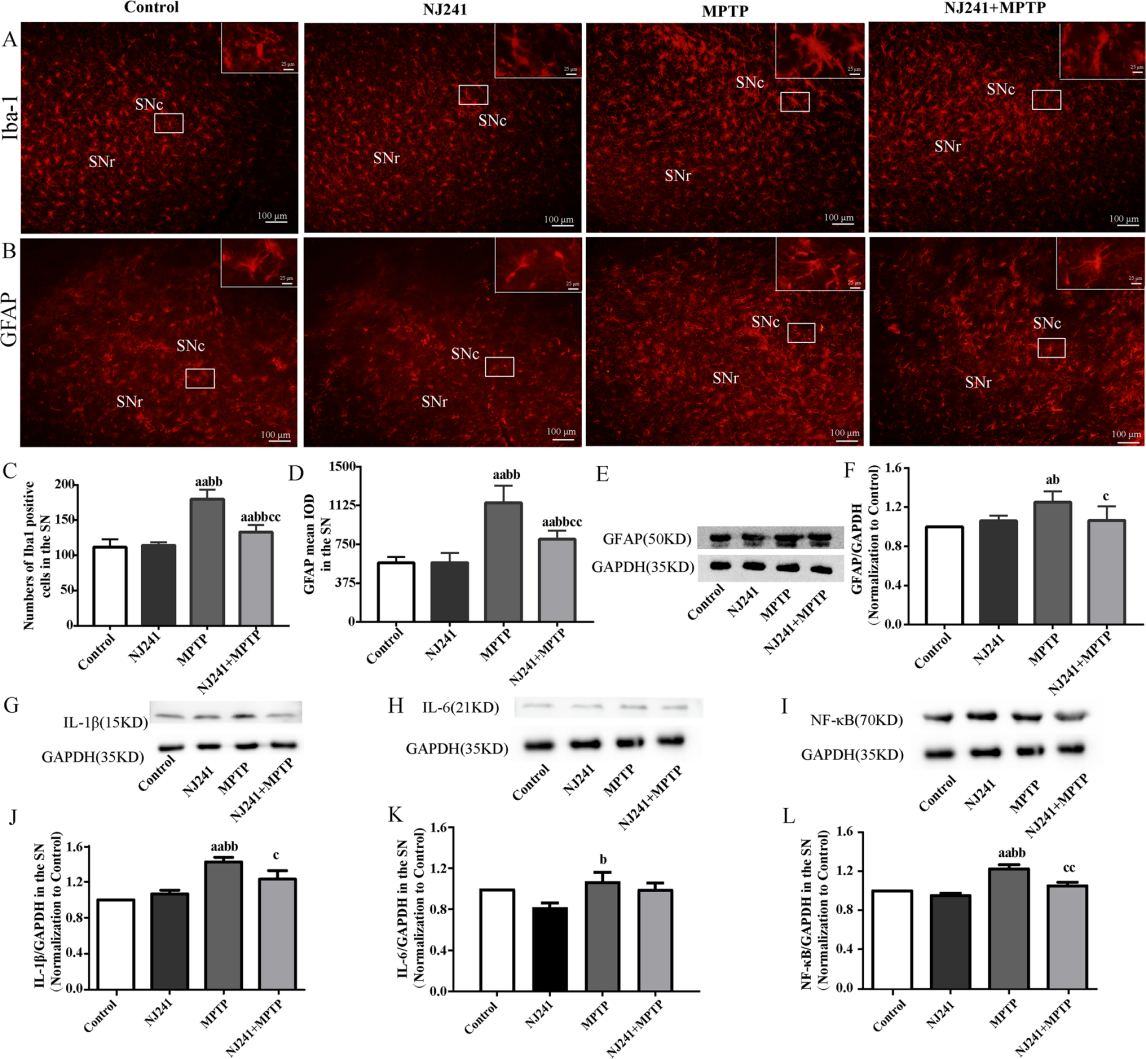
NJ241 inhibits MPTP-induced glial activation in the SN. **A** Iba-1 immunofluorescence showing microglia. **B** GFAP immunofluorescence indicating astrocytes. Scale bars = 100 μm. **C** Quantification of Iba-1 positive cells. **D** Quantification of GFAP fluorescence intensity. **E** Representative GFAP and GAPDH immunoblots. **F** Densitometric analysis of GFAP expression. **G**–**L** Expression levels of inflammatory cytokines IL-1β, IL-6, and NF-κB. *n* = 6 for immunofluorescence, *n* = 3 for immunoblotting. Data are mean ± SEM. SNc, substantia nigra pars compacta; SNr, substantia nigra pars reticulata. A *P* < 0.05, aa *P* < 0.01 vs. control; b *P* < 0.05, bb *P* < 0.01 vs. NJ241; c *P* < 0.05, cc *P* < 0.01 vs. MPTP

Levels of the astrocyte marker GFAP and microglia marker Iba-1 serve as indicators of neuroinflammation severity. We evaluated the impact of NJ241 on glial activation in the SN through immunofluorescence and immunoblotting. No significant differences were observed in Iba-1 positive cells or GFAP immunoreactivity between NJ241 and control mice. Conversely, MPTP-induced mice exhibited heightened Iba-1 positive microglia and increased GFAP fluorescence intensity compared to controls and NJ241-treated group (both* P* < 0.001). NJ241 intervention led to a reduction in Iba-1 and GFAP levels compared to MPTP-induced mice (both *P* < 0.001). Similarly, GFAP protein expression showed no significant difference between NJ241 and control groups but was elevated in MPTP-induced mice compared to controls and NJ241-treated group (*P* = 0.013 and 0.042, respectively). NJ241 treatment resulted in decreased GFAP levels compared to MPTP-induced mice (*P* = 0.045) (Fig. 3A–F).

Sustained astrocyte and microglial activation release inflammatory cytokines such as IL-1β, IL-6, and TNF-α, causing neuronal damage. IL-1β levels did not differ between NJ241 and control groups but were increased in MPTP-induced mice compared to controls and NJ241-treated group (*P* = 0.001 and 0.003). NJ241 + MPTP mice exhibited lower IL-1β levels than MPTP-induced mice (*P* = 0.049). IL-6 was higher in MPTP-induced mice compared to NJ241-treated mice (*P* = 0.014) but showed similar levels between other groups. NF-κB expression was elevated in MPTP-induced mice compared to controls and NJ241-treated mice (both *P* < 0.001) and reduced in NJ241 + MPTP mice compared to MPTP-induced mice (*P* = 0.005) (Fig. 3G–L).

### NJ241 Counteracts MPTP-induced Blood–brain Barrier (BBB) Disruption in the SN

**Fig. 4:**
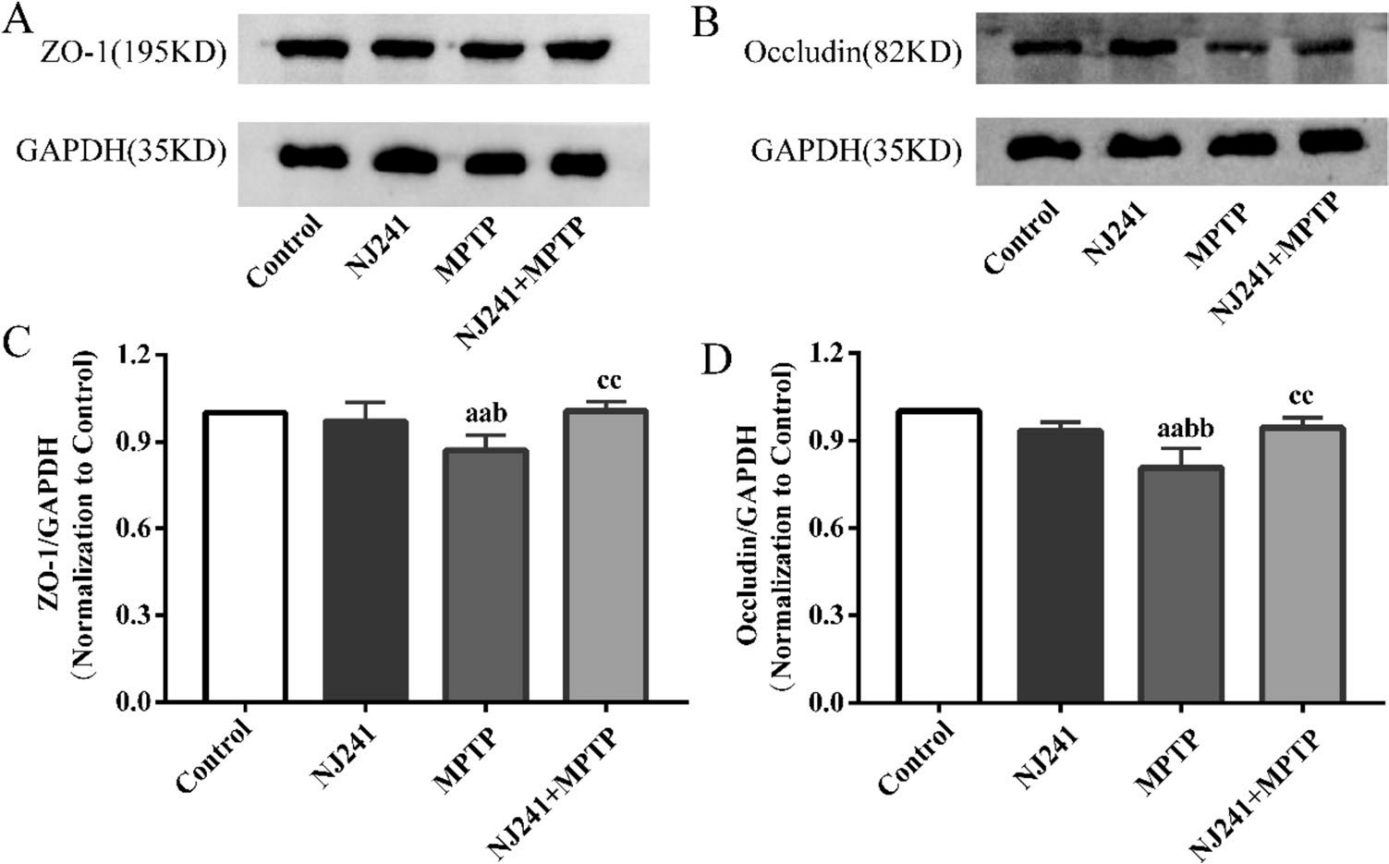
NJ241 protects against MPTP-induced disruption of blood–brain barrier in the SN. **A** Representative ZO-1 and GAPDH immunoblots. **B** Representative occludin and GAPDH immunoblots. **C** Densitometric analysis of ZO-1 expression. **D** Densitometric analysis of occludin expression. *n* = 3 mice/group. Data are mean ± SEM. aa *P* < 0.01 vs. control; b *P* < 0.05, bb *P* < 0.01 vs. NJ241; cc *P* < 0.01 vs. MPTP

The disruption of tight junction proteins, such as ZO-1 and occludin, in the SN leads to elevated BBB permeability, neuroinflammation, and the death of dopaminergic neurons. We evaluated the impact of NJ241 on BBB integrity through the measurement of ZO-1 and occludin expression. ZO-1 levels exhibited no significant differences between NJ241 and control groups. Conversely, MPTP-induced mice demonstrated decreased ZO-1 levels compared to the controls (*P* = 0.008) and the NJ241 group (*P* = 0.026). NJ241 treatment resulted in an increased expression of ZO-1 relative to MPTP-induced mice (*P* = 0.006) (Fig. 4A, C). Similarly, occludin expression showed no significant difference between NJ241 and control groups but was reduced in MPTP-induced mice compared to controls and the NJ241 group (both *P* < 0.001). NJ241 intervention led to an increased occludin expression compared to MPTP-induced mice (*P* = 0.003) (Fig. 4B, D).

### NJ241 Reverses MPTP-induced GM Dysbiosis in Mice

**Fig. 5:**
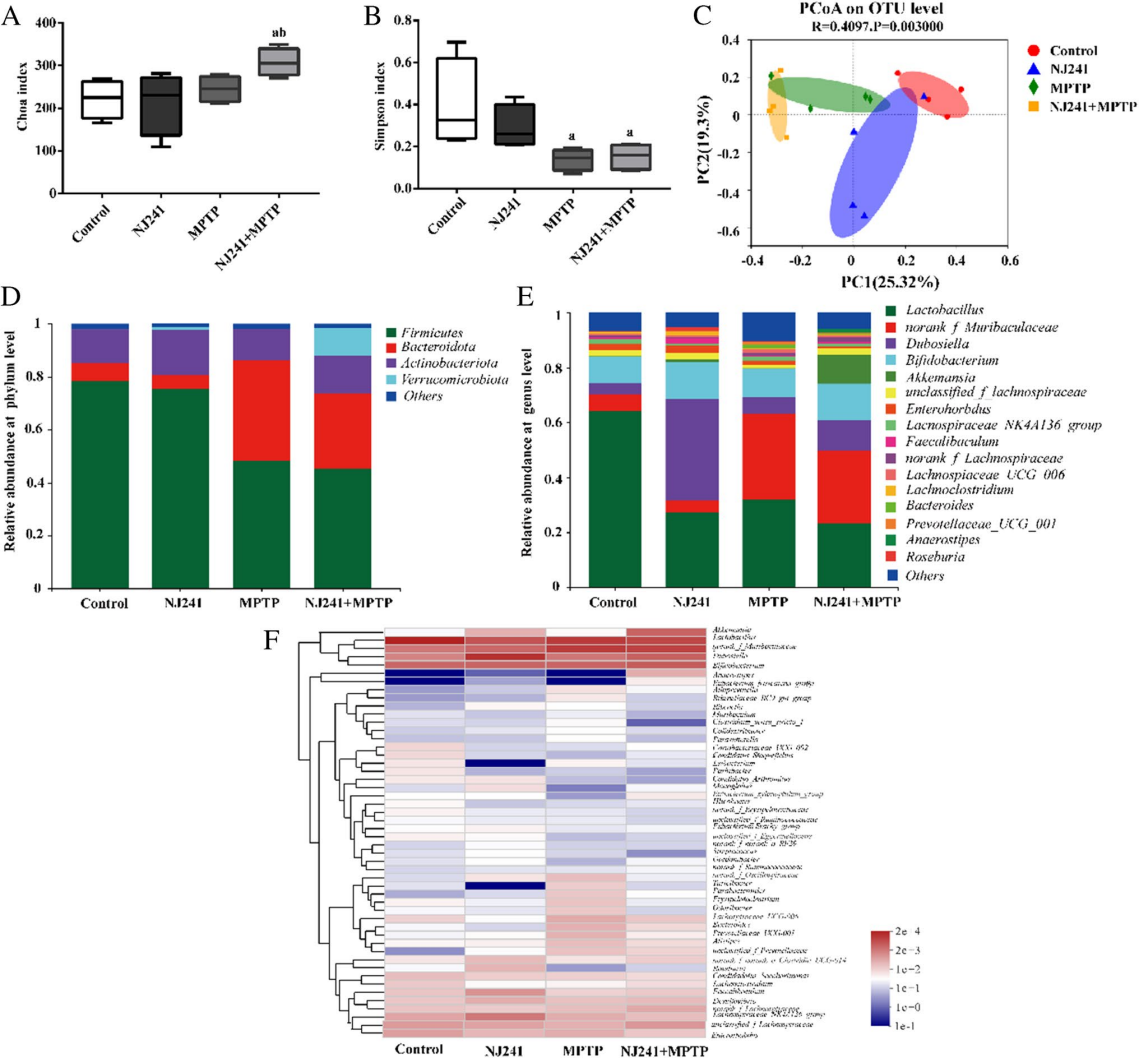
NJ241 improves MPTP-induced alterations in gut microbiota diversity and composition. **A** Chao index showing α-diversity. **B** Simpson index showing α-diversity. **C** PCoA plot displaying β-diversity. **D** Relative abundance at the phylum level. **E** Relative abundance at the genus level. **F** Heatmap of key genus-level changes. a *P* < 0.05 vs. control group; b *P* < 0.05 vs. NJ241 group

To investigate the impact of MPTP on GM and the potential of NJ241 to mitigate any MPTP-induced dysbiosis, we performed 16S rRNA gene sequencing on fecal samples. The Chao index, reflecting richness, exhibited no significant differences between MPTP, control, and NJ241 groups (Fig. 5A). However, the NJ241 + MPTP group demonstrated a higher Chao index compared to control and NJ241 mice (*P* = 0.028 and 0.018). The Simpson index, assessing evenness, decreased in MPTP versus control mice (*P* = 0.013) and in NJ241 + MPTP versus controls (*P* = 0.018), with no difference observed between MPTP and NJ241 + MPTP groups (Fig. 5B). β diversity analysis, measuring between-group differences, showed no distinction between NJ241 and control mice. However, the MPTP group differed significantly from controls, while the NJ241 + MPTP group also diverged from the MPTP mice (Fig. 5C).

Exploring the phylum level (Fig. 5D), a reduction in *Firmicutes* abundance and an increase in *Bacteroidetes* were observed in MPTP versus control and NJ241 groups (*P* = 0.021 and 0.034 for *Firmicutes*; *P* = 0.003 and 0.002 for *Bacteroidetes*). The NJ241 + MPTP group exhibited reduced *Bacteroidetes* and elevated *Verrucomicrobia* compared to MPTP mice. The beneficial bacterium *Lactobacillus* exhibited reduced levels across all groups relative to controls (*P* = 0.02, 0.037, 0.012). *Muribaculaceae* levels were higher in MPTP versus control and NJ241 groups (*P* = 0.008 and 0.005) and displayed a decreasing trend in NJ241 + MPTP mice. Notably, NJ241 groups exhibited heightened levels of the beneficial bacterium *Dubosiella*. Proinflammatory *Bacteroide*s and *Prevotellaceae* were elevated by MPTP but attenuated in NJ241 + MPTP vs. compared to MPTP mice (Fig. 5E).

Heatmap analysis (Fig. 5F) unveiled additional genus-level microbiota alterations. Notably, NJ241 treatment increased potentially beneficial bacteria such as *Akkermansia* while suppressing potentially detrimental bacteria like *Bacteroides* in MPTP-intoxicated mice.

### NJ241 Increases the Levels of SCFAs, Enhances GLP-1 Secretion in Colon, and Upregulates GLP-1R/PGC-1α Expression in the SN

**Fig. 6:**
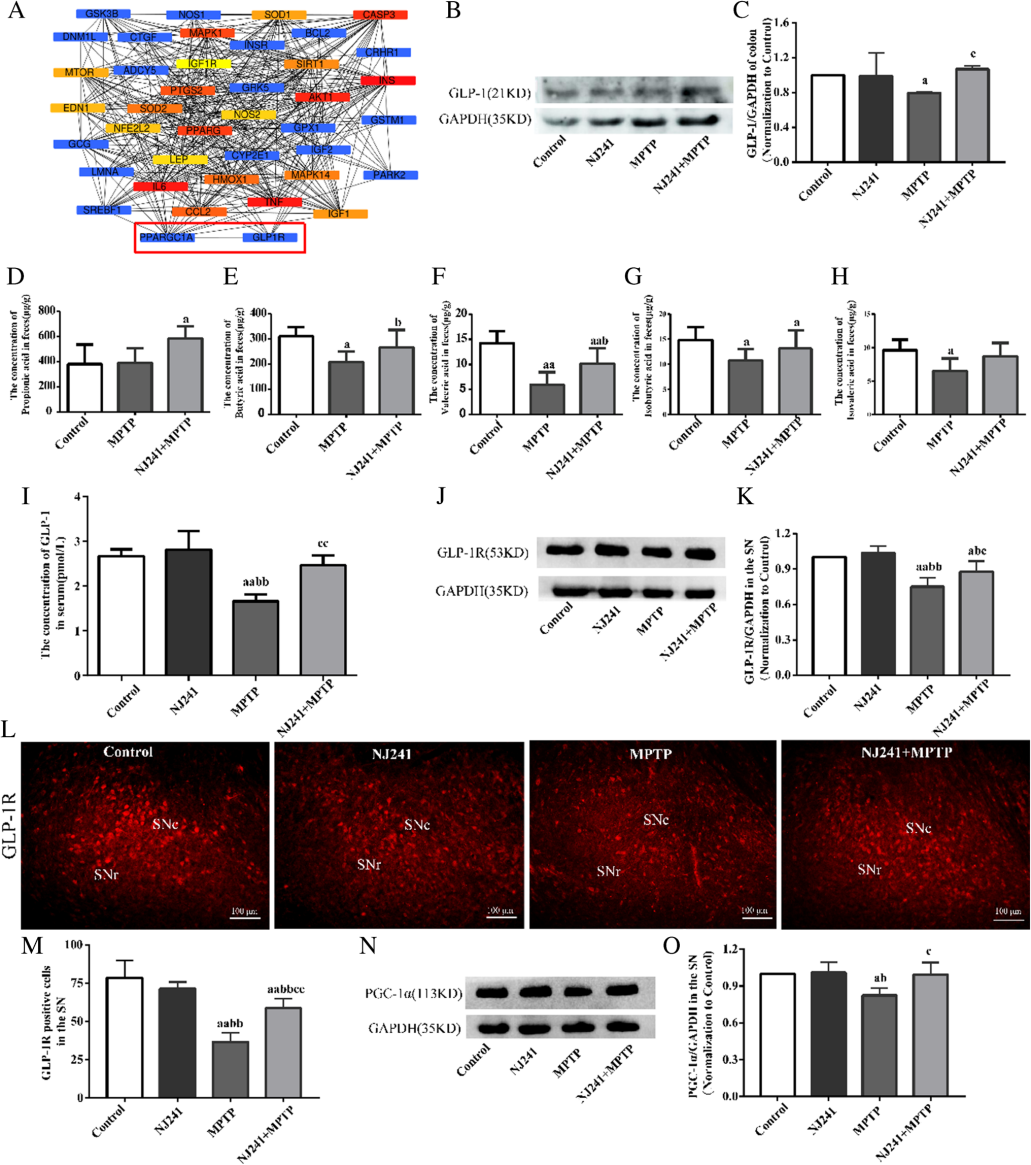
NJ241 restores MPTP-induced decreases in colonic GLP-1, nigral GLP-1R, and PGC-1α. **A** STRING analysis of shared genes in Parkinson’s disease and diabetes. **B**, **C** Colonic GLP-1 protein and quantification. **D**–**H** The analysis of SCFAs in the control group, MPTP group, and NJ241 + MPTP group; **I** serum GLP-1 levels by ELISA. **J**, **K** Nigral GLP-1R protein and quantification. **L**, **M** GLP-1R immunofluorescence and quantification in substantia nigra. **N**, **O** Nigral PGC-1α protein and quantification. *n* = 3–6. Data are mean ± SEM. a *P* < 0.05, aa *P* < 0.01 vs. control; b *P* < 0 0.05, bb *P* < 0.01 vs. NJ241; c *P* < 0.05 vs. MPTP

PD and diabetes exhibit shared chronic, age-related pathogenesis. A query of the DisGeNET database for genes associated with both diseases identified 43 genes. STRING protein–protein interaction network analysis revealed connections between GLP-1R and PGC-1α (Fig. 6A).

GLP-1 is an incretin hormone derived from intestinal L cells. Previous studies have found that intestinal metabolite SCFAs can promote the secretion of GLP-1. In order to detect the relationship between SCFAs and GLP-1, we conducted SCFA detection and intestinal GLP-1 protein detection. The results showed that GLP-1 levels of colonic were reduced in MPTP group compared to control group (*P* = 0.011) but increased in NJ241 + MPTP compared to MPTP mice (*P* = 0.018), normalizing to control levels (Fig. 6B–C).

Analysis of SCFAs in the feces of mice from different experimental groups (control group, MPTP group, and NJ241 + MPTP group) identified six primary SCFAs: acetic acid, propionic acid, butyric acid, valeric acid, isobutyric acid, and isovaleric acid. Acetic acid, propionic acid, and butyric acid emerged as the most abundant among these compounds. Statistical analysis of the SCFA content indicated no significant differences in acetic acid levels across the groups. The propionic acid content in the MPTP group did not exhibit a noteworthy difference compared to the control group (*P* = 0.889). In contrast, NJ241 intervention led to a substantial elevation in propionic acid content relative to the control group (*P* = 0.04). Butyric acid, valeric acid, and isobutyric acid levels were significantly diminished in the MPTP group compared to the control group (butyric acid *P* = 0.021, valeric acid *P* < 0.001, isobutyric acid* P* = 0.042). Conversely, the NJ241 + MPTP group demonstrated a pronounced upregulation of SCFAs in comparison to the MPTP group (butyric acid *P* = 0.049, valeric acid *P* = 0.024, isobutyric acid *P* = 0.041). Isovaleric acid exhibited a notable decrease in the MPTP group (*P* = 0.021). However, NJ241 intervention displayed an increasing trend in its expression in PD mice, although the difference did not reach statistical significance when compared to the MPTP group (*P* = 0.104) (Fig. 6D–H).

Then serum GLP-1 concentrations, measured by ELISA, were lower in MPTP compared to  control and NJ241 groups (both *P* < 0.001) and elevated in NJ241 + MPTP compared to MPTP mice (*P* = 0.001, Fig. 6I). Previous study suggested that GLP-1 can traverse the BBB to exert its effects on central GLP-1R. GLP-1R expressions in the SN were measured through immunofluorescence and immunoblotting. Nigral GLP-1R protein and immunopositive cell numbers exhibited no significant differences between NJ241 and control groups. However, MPTP mice demonstrated decreased GLP-1R protein and cell counts relative to controls (*P* = 0.002 and *P* < 0.001, respectively) and the NJ241 group (*P* = 0.001 for both measures). Notably, NJ241 treatment augmented nigral GLP-1R protein and immunofluorescence compared to MPTP mice (*P* = 0.044 and *P* < 0.001, Fig. 6J–M).

Finally, nigral PGC-1α expression was diminished in MPTP compared to control and NJ241 mice (*P* = 0.015 and 0.012) but increased in NJ241 + MPTP compared to MPTP mice (*P* = 0.018, Fig. 6N–O).

### GLP-1R Antagonists Partially Reverse the Inhibition of Glial Cell Activation by NJ241

**Fig. 7:**
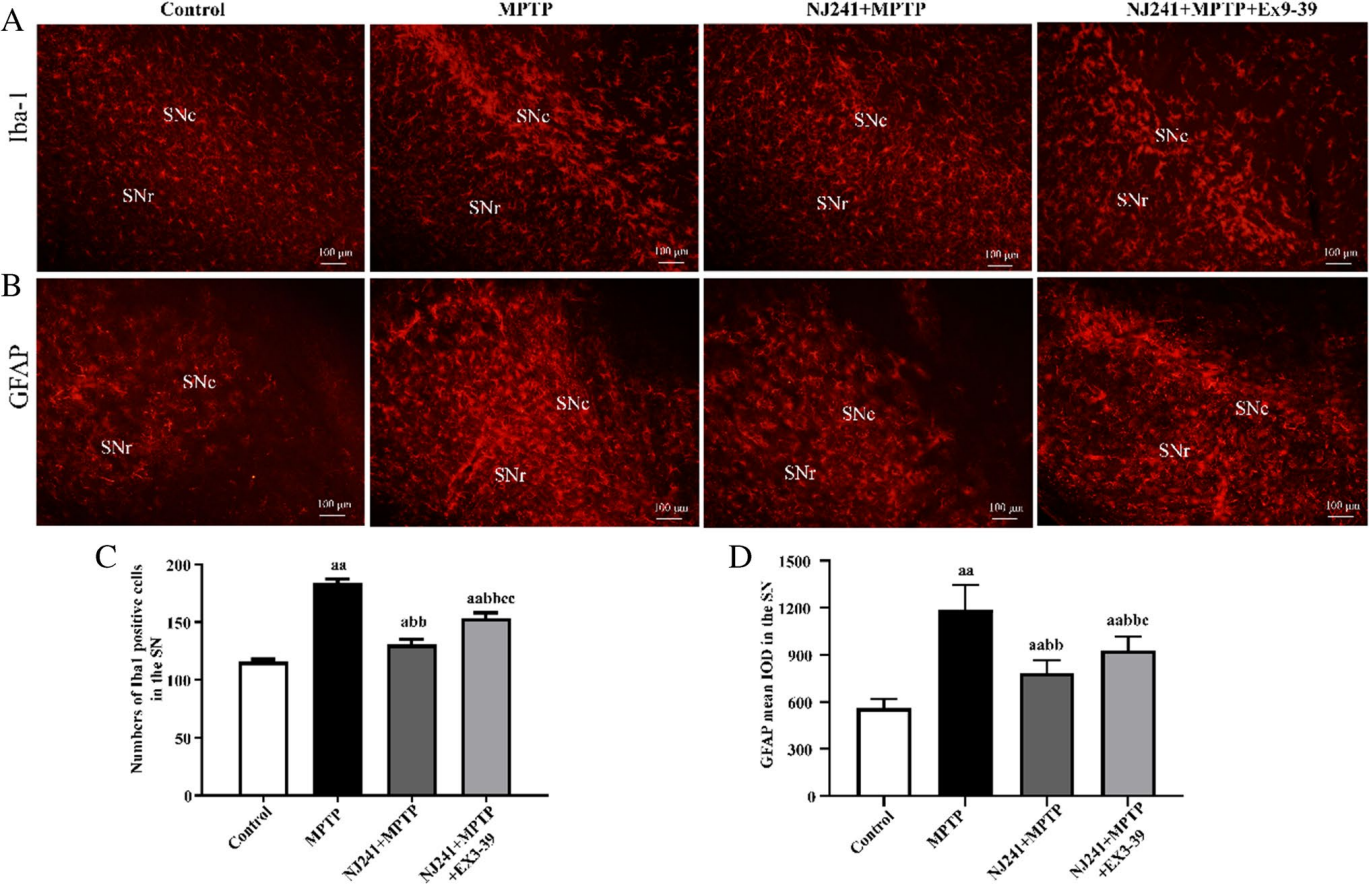
GLP-1R antagonists reversed the inhibition of glial cell activation by NJ241. **A** Iba-1 immunofluorescence showing microglia. **B** GFAP immunofluorescence indicating astrocytes. Scale bars = 100 μm. **C** Quantification of Iba-1 positive cells. **D** Quantification of GFAP fluorescence intensity. *n* = 5. Data are mean ± SEM. a *P* < 0.05, aa *P* < 0.01 vs. control; bb *P* < 0.01 vs. NJ241; **c** *P* < 0.05, cc* P* < 0.01 vs. NJ241 + MPTP

To ascertain whether NJ241 mitigates neuroinflammation in the SN through the mediation of GLP-1 on GLP-1R, we employed the GLP-1R antagonist Ex9-39 to attenuate the impact of GLP-1R, subsequently assessing inflammation in the SN via immunofluorescence. The results revealed that Ex9-39 reversed the inhibitory effects of NJ241 on microglial and astrocyte activation. Microglial activation was significantly higher in the Ex9-39 group compared to the NJ241 + MPTP group (*P* < 0.001). Moreover, astrocyte activation demonstrated a marked increase relative to the NJ241 + MPTP group (*P* = 0.044). Additionally, the count of TH-positive cells was substantially lower in the Ex9-39 group compared to the NJ241 + MPTP group (*P* = 0.006). However, no statistically significant difference was observed between the Ex9-39 and MPTP groups (*P* = 0.207) (Fig. 7).

## Discussion

In recent studies, a compelling body of evidence has emerged, establishing robust connections between dysbiosis in the GM and the onset and progression of PD. Notably, the probiotic *Bifidobacterium animalis* has been identified as a pivotal gut microbe in these associations. Some investigations propose that *Bifidobacterium animalis* may exert neuroprotective and neuroreparative effects in PD. For instance, a 3-month double-blind placebo-controlled trial demonstrated that the *Bifidobacterium animalis subsp. lactis* strain Probio-M8 ameliorated sleep disturbances, anxiety, gastrointestinal symptoms, and enhanced the clinical efficacy of conventional Parkinson’s medications [26]. These promising results suggest that *Bifidobacterium animalis* holds potential as a microbial therapeutic for PD, though further research in this area is imperative.

In our study, we investigated whether the *Bifidobacterium animalis subsp*. *lactis* strain NJ241 could confer protection against PD. Utilizing immunohistochemistry and immunoblotting, we observed that NJ241 alleviated motor deficits, increased the number of dopaminergic neurons, and enhanced the expression of TH. Moreover, it suppressed glial activation and prevented BBB disruption in the SN of Parkinson’s model mice. NJ241 also rectified GM dysbiosis induced by neurotoxin, reducing proinflammatory taxa while restoring beneficial microbes such as *Bifidobacterium*. Additionally, NJ241 increased feces SCFA content and colonic GLP-1 and enhanced GLP-1R/PGC-1α signaling in the SN. The inhibition of GLP-1R reversed the attenuating effects of NJ241 on nigral glial activation. In summary, our results indicate that NJ241 promotes the production of intestinal GLP-1, which then acts through central GLP-1R/PGC-1α to protect against the pathogenesis of PD.

Substantial evidence from human samples and animal models supports the pivotal role of inflammation in the onset and progression of PD [27]. Elevated levels of proinflammatory cytokines are observed in the cerebrospinal fluid and blood of PD patients. Activated microglia are also detected in the SNc [28]. In the central nervous system, microglia and astrocytes function as innate immune cells, exhibiting both detrimental and beneficial effects in neurodegenerative conditions. Prolonged activation of these glial cells can induce the release of inflammatory cytokines and reactive oxygen species, fostering neurotoxic inflammation [29, 30]. This process disrupts the BBB, allowing the influx of peripheral immune cells such as monocytes, macrophages, neutrophils, and T cells, further amplifying central inflammation [29, 30]. In our study, the neurotoxin MPTP induced pronounced microglial and astroglial activation in the SN, while NJ241 treatment significantly mitigated glial activation. These results align with other probiotic studies in neuroinflammatory contexts [11, 31–33]. NJ241 also alleviated MPTP-induced BBB disruption, aligning with probiotic effects on the barrier observed in other diseases [34–36]. Additionally, a combination of *Bifidobacterium infants* and *Lactobacillus acidophilus* was reported to alleviate abdominal pain and diarrhea in PD patients [37].

Clinically, Parkinson’s patients frequently exhibit gastrointestinal disturbances and weight loss [38, 39]. In our study, MPTP induced weight loss in mice, while NJ241 reversed this effect. Employing an Evans blue dye motility assay, we found that MPTP reduced intestinal transit distance over a defined time period, whereas NJ241 increased motility. Similar results were observed in a rotenone-induced Parkinson’s model after fecal microbiota transplantation, which alleviated weight loss and intestinal dysfunction [35]. Furthermore, a randomized controlled trial by Ibrahim et al. demonstrated that a multi-strain probiotic improved constipation and intestinal motility in PD patients [40].

PD patients frequently manifest gastrointestinal disturbances preceding motor dysfunction, suggesting the involvement of GM in early pathogenesis [41–44]. Thus, probiotic supplementation emerges as a potential therapeutic avenue. Employing 16S rRNA sequencing, we found that the probiotic *Bifidobacterium animalis subsp. lactis* NJ241 mitigated MPTP-induced increases in putative pathobionts such as *Turicibacter*, *Oscillospiraceae*, *Parabacteroides*, *Erysipelatoclostridium*, and *Odoribacte*. Concurrently, NJ241 elevated potentially beneficial *Roseburia* and *Akkermansia*. NJ241 intervention to reduce the inflammatory microbiota and increase the beneficial microbiota may positively affect the gut microbiota by affecting the host’s immune system, enhancing intestinal barrier function, and reducing transmembrane transfer of pathogens and endotoxins. While the specific microbiota alterations differed from other human and animal studies, the overall impact of NJ241 was a reduction in proinflammatory taxa and restoration of anti-inflammatory bacteria. Discrepancies across studies may stem from variations in geographical region, diet, environment, animal models and species, and the stage of PD during fecal sampling [45–47]. Nevertheless, our results aligned with the general trend of microbiota-induced neuroinflammation in PD.

Although the GM evidently regulates the progression of neurodegenerative diseases, the precise molecular mechanisms underlying microbiota-host interactions remain elusive. GLP-1, an intestinally derived peptide hormone, plays a crucial role in modulating the gut-brain axis and energy metabolism, demonstrating neurotrophic and neuroprotective properties [48, 49]. PGC-1α, a master regulator of mitochondrial biogenesis and various metabolic and antioxidant pathways, is implicated in Parkinson’s pathogenesis, with upregulation proving neuroprotective [50, 51]. Our previous research indicated that the GLP-1R agonist liraglutide inhibits neuroinflammation through AMPK/PGC-1α signaling, enhancing mitochondrial quality control via PGC-1α to protect dopaminergic neurons [19, 52].

Relevant probiotic studies have shown that VSL#3 stimulates GLP-1 release, reducing food intake, improving glucose tolerance, and alleviating nonalcoholic fatty liver disease in children via enhanced GLP-1 [51]. An engineered GLP-1-secreting probiotic strain reduced pathogenic Enterobacteriaceae, increased beneficial *Lactobacillus* and *Akkermansia*, and ameliorated MPTP-induced movement deficits, dopaminergic neuron loss, and glial activation [17]. Evaluation of 14 probiotics in diabetic mice revealed anti-hyperglycemic effects involved increased SCFA-producing bacteria, improved intestinal barrier function, and upregulated GLP-1 [53]. While Sampson [54] highlighted the deleterious effects of SCFAs in PD models, other studies have presented contrasting evidence, demonstrating the potential benefits of SCFAs in PD patients or animal models with diminished SCFA content [55–57]. Interventions involving specific SCFAs have been shown to alleviate PD symptoms [58]. Our results showed no significant differences in acetic acid levels among groups, but some bacteria require acetic acid to survive. For example, the butyric-producing bacterium *Coprofecalis prevotelli* cannot grow in a pure culture without acetic acid. Acetic acid plays an important role in butyricogenic bacteria. Our results indicate a reduction in butyric acid, valeric acid, isobutyric acid due to MPTP treatment, and NJ241 intervention effectively reversed this alteration, aligning with previous research affirming the benefits of SCFAs. Clostridium butyrate producers were shown to improve MPTP-induced motor dysfunction, neurodegeneration, synaptopathy, and neuroinflammation while normalizing gut dysbiosis and restoring decreased colonic GLP-1, GPR41/43, and brain GLP-1R in mice [41]. Similarly, we observed that MPTP reduced colonic GLP-1 and nigral GLP-1R/PGC-1α, while NJ241 reversed these effects. Collectively, these results suggest that *Bifidobacterium animalis subsp. lactis* NJ241 may stimulate intestinal GLP-1 production, which subsequently acts via nigral GLP-1R/PGC-1α signaling to exert neuroprotection in PD.

This study enhances our understanding of probiotic regulation of neuroinflammation and its therapeutic potential in PD, contributing to the knowledge of these natural bioactive compounds. Nevertheless, limitations exist, including the use of only one PD mouse model, which cannot fully simulate the complex pathological mechanism of human PD; the use of only one probiotic strain, which cannot reflect the diversity and interaction of intestinal microbiota; observation of only some changes in genes and neurotransmitters, without exploring the specific molecular mechanism of the PGC-1α signaling pathway; and a 4-week experimental duration, without considering the long-term effects and safety. As research progresses, further insights will be gained into the applications of probiotics in neurodegenerative diseases.

## Data Availability

Data can be made available by contacting the corresponding author.

## References

[1] Li G, Ma J, Cui S, He Y, Xiao Q, Liu J, Chen S (2019) Parkinson’s disease in China: a forty-year growing track of bedside work. Transl Neurodegener 8:22. 10.1186/s40035-019-0162-z 31384434 10.1186/s40035-019-0162-z PMC6668186

[2] Braak H, de Vos RA, Bohl J, Del Tredici K (2006) Gastric alpha-synuclein immunoreactive inclusions in Meissner’s and Auerbach’s plexuses in cases staged for Parkinson’s disease-related brain pathology. Neurosci Lett 396(1):67–72. 10.1016/j.neulet.2005.11.012 16330147 10.1016/j.neulet.2005.11.012

[3] Holmqvist S, Chutna O, Bousset L, Aldrin-Kirk P, Li W, Björklund T, Wang ZY, Roybon L et al (2014) Direct evidence of Parkinson pathology spread from the gastrointestinal tract to the brain in rats. Acta Neuropathol 128(6):805–820. 10.1007/s00401-014-1343-6 25296989 10.1007/s00401-014-1343-6

[4] Lee HS, Lobbestael E, Vermeire S, Sabino J, Cleynen I (2021) Inflammatory bowel disease and Parkinson’s disease: common pathophysiological links. Gut 70(2):408–417. 10.1136/gutjnl-2020-322429 33067333 10.1136/gutjnl-2020-322429

[5] Parashar A, Udayabanu M (2017) Gut microbiota: implications in Parkinson’s disease. Parkinsonism Relat Disord 38:1–7. 10.1016/j.parkreldis.2017.02.002 28202372 10.1016/j.parkreldis.2017.02.002 PMC7108450

[6] Nimgampalle M, Kuna Y (2017) Anti-Alzheimer properties of probiotic, Lactobacillus plantarum MTCC 1325 in Alzheimer’s disease induced albino rats. J Clin Diagn Res 11(8):KC01–KC05. 10.7860/JCDR/2017/26106.10428 28969160 10.7860/JCDR/2017/26106.10428 PMC5620801

[7] Wasser CI, Mercieca EC, Kong G, Hannan AJ, McKeown SJ, Glikmann-Johnston Y, Stout JC (2020) Gut dysbiosis in Huntington’s disease: associations among gut microbiota, cognitive performance and clinical outcomes. Brain Commun 2(2):fcaa110. 10.1093/braincomms/fcaa110 33005892 10.1093/braincomms/fcaa110 PMC7519724

[8] Leblhuber F, Ehrlich D, Steiner K, Geisler S, Fuchs D, Lanser L, Kurz K (2021) The immunopathogenesis of Alzheimer’s disease is related to the composition of gut microbiota. Nutrients 13(2). 10.3390/nu13020361 10.3390/nu13020361 PMC7912578 33504065

[9] Martorell P, Alvarez B, Llopis S, Navarro V, Ortiz P, Gonzalez N, Balaguer F, Rojas A, et al. (2021) Heat-treated Bifidobacterium longum CECT-7347: a whole-cell postbiotic with antioxidant, anti-inflammatory, and gut-barrier protection properties. Antioxidants (Basel) 10(4). 10.3390/antiox10040536 10.3390/antiox10040536 PMC8067082 33808122

[10] Labarre A, Guitard E, Tossing G, Forest A, Bareke E, Labrecque M, Tétreault M, Ruiz M et al (2022) Fatty acids derived from the probiotic Lacticaseibacillus rhamnosus HA-114 suppress age-dependent neurodegeneration. Commun Biol 5(1):1340. 10.1038/s42003-022-04295-8 36477191 10.1038/s42003-022-04295-8 PMC9729297

[11] Wang L, Zhao Z, Zhao L, Zhao Y, Yang G, Wang C, Gao L, Niu C et al (2022) Lactobacillus plantarum DP189 reduces α-SYN aggravation in MPTP-induced Parkinson’s disease mice via regulating oxidative damage, inflammation, and gut microbiota disorder. J Agric Food Chem 70(4):1163–1173. 10.1021/acs.jafc.1c07711 35067061 10.1021/acs.jafc.1c07711

[12] Macfarlane GT, Macfarlane S (2012) Bacteria, colonic fermentation, and gastrointestinal health. J AOAC Int 95(1):50–60. 10.5740/jaoacint.sge_macfarlane 22468341 10.5740/jaoacint.sge_macfarlane

[13] Caspani G, Swann J (2019) Small talk: microbial metabolites involved in the signaling from microbiota to brain. Curr Opin Pharmacol 48:99–106. 10.1016/j.coph.2019.08.001 31525562 10.1016/j.coph.2019.08.001

[14] Liu J, Wang F, Liu S, Du J, Hu X, Xiong J, Fang R, Chen W et al (2017) Sodium butyrate exerts protective effect against Parkinson’s disease in mice via stimulation of glucagon like peptide-1. J Neurol Sci 381:176–181. 10.1016/j.jns.2017.08.3235 28991675 10.1016/j.jns.2017.08.3235

[15] Cork SC, Richards JE, Holt MK, Gribble FM, Reimann F, Trapp S (2015) Distribution and characterisation of glucagon-like peptide-1 receptor expressing cells in the mouse brain. Mol Metab 4(10):718–731. 10.1016/j.molmet.2015.07.008 26500843 10.1016/j.molmet.2015.07.008 PMC4588458

[16] Batista AF, Bodart-Santos V, De Felice FG, Ferreira ST (2019) Neuroprotective actions of glucagon-like peptide-1 (GLP-1) analogues in Alzheimer’s and Parkinson’s diseases. CNS Drugs 33(3):209–223. 10.1007/s40263-018-0593-6 30511349 10.1007/s40263-018-0593-6

[17] Fang X, Tian P, Zhao X, Jiang C, Chen T (2019) Neuroprotective effects of an engineered commensal bacterium in the 1-methyl-4-phenyl-1, 2, 3, 6-tetrahydropyridine Parkinson disease mouse model via producing glucagon-like peptide-1. J Neurochem 150(4):441–452. 10.1111/jnc.14694 30851189 10.1111/jnc.14694

[18] Chen YY, Yan Y, Zhao Z, Shi MJ, Zhang YB (2016) Bofutsushosan ameliorates obesity in mice through modulating PGC-1α expression in brown adipose tissues and inhibiting inflammation in white adipose tissues. Chin J Nat Med 14(6):449–456. 10.1016/S1875-5364(16)30042-5 27473963 10.1016/S1875-5364(16)30042-5

[19] Cao B, Zhang Y, Chen J, Wu P, Dong Y, Wang Y (2022) Neuroprotective effects of liraglutide against inflammation through the AMPK/NF-κB pathway in a mouse model of Parkinson’s disease. Metab Brain Dis 37(2):451–462. 10.1007/s11011-021-00879-1 34817756 10.1007/s11011-021-00879-1

[20] Hu F, Duan M, Peng N (2019) Knockdown of TRB3 improved the MPP(+)/MPTP-induced Parkinson’s disease through the MAPK and AKT signaling pathways. Neurosci Lett 709:134352. 10.1016/j.neulet.2019.134352 31283965 10.1016/j.neulet.2019.134352

[21] Edgar RC (2013) UPARSE: highly accurate OTU sequences from microbial amplicon reads. Nat Methods 10(10):996–998. 10.1038/nmeth.2604 23955772 10.1038/nmeth.2604

[22] Wang Q, Garrity GM, Tiedje JM, Cole JR (2007) Naive Bayesian classifier for rapid assignment of rRNA sequences into the new bacterial taxonomy. Appl Environ Microbiol 73(16):5261–5267. 10.1128/AEM.00062-07 17586664 10.1128/AEM.00062-07 PMC1950982

[23] Han X, Guo J, You Y, Yin M, Ren C, Zhan J, Huang W (2018) A fast and accurate way to determine short chain fatty acids in mouse feces based on GC-MS. J Chromatogr B Analyt Technol Biomed Life Sci 1099:73–82. 10.1016/j.jchromb.2018.09.013 30243116 10.1016/j.jchromb.2018.09.013

[24] Zhang S, Wang H, Zhu MJ (2019) A sensitive GC/MS detection method for analyzing microbial metabolites short chain fatty acids in fecal and serum samples. Talanta 196:249–254. 10.1016/j.talanta.2018.12.049 30683360 10.1016/j.talanta.2018.12.049

[25] Hsu YL, Chen CC, Lin YT, Wu WK, Chang LC, Lai CH, Wu MS, Kuo CH (2019) Evaluation and optimization of sample handling methods for quantification of short-chain fatty acids in human fecal samples by GC-MS. J Proteome Res 18(5):1948–1957. 10.1021/acs.jproteome.8b00536 30895795 10.1021/acs.jproteome.8b00536

[26] Sun H, Zhao F, Liu Y, Ma T, Jin H, Quan K, Leng B, Zhao J et al (2022) Probiotics synergized with conventional regimen in managing Parkinson’s disease. NPJ Parkinsons Dis 8(1):62. 10.1038/s41531-022-00327-6 35610236 10.1038/s41531-022-00327-6 PMC9130297

[27] Tansey MG, Wallings RL, Houser MC, Herrick MK, Keating CE, Joers V (2022) Inflammation and immune dysfunction in Parkinson disease. Nat Rev Immunol 22(11):657–673. 10.1038/s41577-022-00684-6 35246670 10.1038/s41577-022-00684-6 PMC8895080

[28] Nagatsu T, Mogi M, Ichinose H, Togari A (2000) Changes in cytokines and neurotrophins in Parkinson’s disease. J Neural Transm Suppl 60:277–290. 10.1007/978-3-7091-6301-6_19 10.1007/978-3-7091-6301-6_19 11205147

[29] Kumar V (2019) Toll-like receptors in the pathogenesis of neuroinflammation. J Neuroimmunol 332:16–30. 10.1016/j.jneuroim.2019.03.012 30928868 10.1016/j.jneuroim.2019.03.012

[30] Heidari A, Rostam-Abadi Y, Rezaei N (2021) The immune system and autism spectrum disorder: association and therapeutic challenges. Acta Neurobiol Exp (Wars) 81(3):249–263. 10.21307/ane-2021-023 34672295 10.21307/ane-2021-023

[31] Liao JF, Cheng YF, You ST, Kuo WC, Huang CW, Chiou JJ, Hsu CC, Hsieh-Li HM et al (2020) Lactobacillus plantarum PS128 alleviates neurodegenerative progression in 1-methyl-4-phenyl-1,2,3,6-tetrahydropyridine-induced mouse models of Parkinson’s disease. Brain Behav Immun 90:26–46. 10.1016/j.bbi.2020.07.036 32739365 10.1016/j.bbi.2020.07.036

[32] Li T, Chu C, Yu L, Zhai Q, Wang S, Zhao J, Zhang H, Chen W, et al. (2022) Neuroprotective effects of Bifidobacterium breve CCFM1067 in MPTP-induced mouse models of Parkinson’s disease. Nutrients 14(21). 10.3390/nu14214678 10.3390/nu14214678 PMC9655354 36364939

[33] Chu C, Yu L, Li Y, Guo H, Zhai Q, Chen W, Tian F (2023) Lactobacillus plantarum CCFM405 against rotenone-induced Parkinson’s disease mice via regulating gut microbiota and branched-chain amino acids biosynthesis. Nutrients 15(7):1737. 10.3390/nu15071737 37049578 10.3390/nu15071737 PMC10096885

[34] Yang X, Yu D, Xue L, Li H, Du J (2020) Probiotics modulate the microbiota-gut-brain axis and improve memory deficits in aged SAMP8 mice. Acta Pharm Sin B 10(3):475–487. 10.1016/j.apsb.2019.07.001 32140393 10.1016/j.apsb.2019.07.001 PMC7049608

[35] Zhao Z, Ning J, Bao XQ, Shang M, Ma J, Li G, Zhang D (2021) Fecal microbiota transplantation protects rotenone-induced Parkinson’s disease mice via suppressing inflammation mediated by the lipopolysaccharide-TLR4 signaling pathway through the microbiota-gut-brain axis. Microbiome 9(1):226. 10.1186/s40168-021-01107-9 34784980 10.1186/s40168-021-01107-9 PMC8597301

[36] Lu J, Fan X, Lu L, Yu Y, Markiewicz E, Little JC, Sidebottom AM, Claud EC (2023) Limosilactobacillus reuteri normalizes blood-brain barrier dysfunction and neurodevelopment deficits associated with prenatal exposure to lipopolysaccharide. Gut Microbes 15(1):2178800. 10.1080/19490976.2023.2178800 36799469 10.1080/19490976.2023.2178800 PMC9980478

[37] Georgescu D, Ancusa OE, Georgescu LA, Ionita I, Reisz D (2016) Nonmotor gastrointestinal disorders in older patients with Parkinson’s disease: is there hope. Clin Interv Aging 11:1601–1608. 10.2147/CIA.S106284 27956826 10.2147/CIA.S106284 PMC5113937

[38] Fasano A, Visanji NP, Liu LW, Lang AE, Pfeiffer RF (2015) Gastrointestinal dysfunction in Parkinson’s disease. Lancet Neurol 14(6):625–639. 10.1016/S1474-4422(15)00007-1 25987282 10.1016/S1474-4422(15)00007-1

[39] De Rui M, Inelmen EM, Trevisan C, Pigozzo S, Manzato E, Sergi G (2020) Parkinson’s disease and the non-motor symptoms: hyposmia, weight loss, osteosarcopenia. Aging Clin Exp Res 32(7):1211–1218. 10.1007/s40520-020-01470-x 31989535 10.1007/s40520-020-01470-x

[40] Ibrahim A, Ali R, Manaf M, Ahmad N, Tajurruddin FW, Qin WZ, Desa S, Ibrahim NM (2020) Multi-strain probiotics (Hexbio) containing MCP BCMC strains improved constipation and gut motility in Parkinson’s disease: a randomised controlled trial. PLoS One 15(12):e0244680. 10.1371/journal.pone.0244680 33382780 10.1371/journal.pone.0244680 PMC7774928

[41] Scheperjans F, Aho V, Pereira PA, Koskinen K, Paulin L, Pekkonen E, Haapaniemi E, Kaakkola S et al (2015) Gut microbiota are related to Parkinson’s disease and clinical phenotype. Mov Disord 30(3):350–358. 10.1002/mds.26069 25476529 10.1002/mds.26069

[42] Sun MF, Zhu YL, Zhou ZL, Jia XB, Xu YD, Yang Q, Cui C, Shen YQ (2018) Neuroprotective effects of fecal microbiota transplantation on MPTP-induced Parkinson’s disease mice: gut microbiota, glial reaction and TLR4/TNF-α signaling pathway. Brain Behav Immun 70:48–60. 10.1016/j.bbi.2018.02.005 29471030 10.1016/j.bbi.2018.02.005

[43] Li F, Wang P, Chen Z, Sui X, Xie X, Zhang J (2019) Alteration of the fecal microbiota in North-Eastern Han Chinese population with sporadic Parkinson’s disease. Neurosci Lett 707:134297. 10.1016/j.neulet.2019.134297 31200089 10.1016/j.neulet.2019.134297

[44] Zhao Z, Li F, Ning J, Peng R, Shang J, Liu H, Shang M, Bao XQ et al (2021) Novel compound FLZ alleviates rotenone-induced PD mouse model by suppressing TLR4/MyD88/NF-κB pathway through microbiota-gut-brain axis. Acta Pharm Sin B 11(9):2859–2879. 10.1016/j.apsb.2021.03.020 34589401 10.1016/j.apsb.2021.03.020 PMC8463266

[45] Weis S, Schwiertz A, Unger MM, Becker A, Faßbender K, Ratering S, Kohl M, Schnell S et al (2019) Effect of Parkinson’s disease and related medications on the composition of the fecal bacterial microbiota. NPJ Parkinsons Dis 5:28. 10.1038/s41531-019-0100-x 31815177 10.1038/s41531-019-0100-x PMC6884491

[46] Zhang F, Yue L, Fang X, Wang G, Li C, Sun X, Jia X, Yang J et al (2020) Altered gut microbiota in Parkinson’s disease patients/healthy spouses and its association with clinical features. Parkinsonism Relat Disord 81:84–88. 10.1016/j.parkreldis.2020.10.034 33099131 10.1016/j.parkreldis.2020.10.034

[47] Nie S, Wang J, Deng Y, Ye Z, Ge Y (2022) Inflammatory microbes and genes as potential biomarkers of Parkinson’s disease. NPJ Biofilms Microbiomes 8(1):101. 10.1038/s41522-022-00367-z 36564391 10.1038/s41522-022-00367-z PMC9789082

[48] Hölscher C (2012) Potential role of glucagon-like peptide-1 (GLP-1) in neuroprotection. CNS Drugs 26(10):871–882. 10.2165/11635890-000000000-00000 22938097 10.2165/11635890-000000000-00000

[49] Ammar RA, Mohamed AF, Kamal MM, Safar MM, Abdelkader NF (2022) Neuroprotective effect of liraglutide in an experimental mouse model of multiple sclerosis: role of AMPK/SIRT1 signaling and NLRP3 inflammasome. Inflammopharmacology 30(3):919–934. 10.1007/s10787-022-00956-6 35364735 10.1007/s10787-022-00956-6 PMC9135867

[50] Kang H, Khang R, Ham S, Jeong GR, Kim H, Jo M, Lee BD, Lee YI et al (2017) Activation of the ATF2/CREB-PGC-1α pathway by metformin leads to dopaminergic neuroprotection. Oncotarget 8(30):48603–48618. 10.18632/oncotarget.18122 28611284 10.18632/oncotarget.18122 PMC5564711

[51] Ma D, Liu X, Liu J, Li M, Chen L, Gao M, Xu W, Yang Y (2019) Long-term liraglutide meliorates nigrostriatal impairment via regulating AMPK/PGC-1a signaling in diabetic mice. Brain Res 1714:126–132. 10.1016/j.brainres.2019.02.030 30826352 10.1016/j.brainres.2019.02.030

[52] Wu PY, Dong YX, Chen JH, Guan TY, Cao B, Zhang YQ, Qi YY, Guan ZL et al (2022) Liraglutide regulates mitochondrial quality control system through PGC-1α in a mouse model of Parkinson’s disease. Neurotox Res 40(1):286–297. 10.1007/s12640-021-00460-9 35043376 10.1007/s12640-021-00460-9

[53] Wang Y, Dilidaxi D, Wu Y, Sailike J, Sun X, Nabi XH (2020) Composite probiotics alleviate type 2 diabetes by regulating intestinal microbiota and inducing GLP-1 secretion in db/db mice. Biomed Pharmacother 125:109914. 10.1016/j.biopha.2020.109914 32035395 10.1016/j.biopha.2020.109914

[54] Sampson TR, Debelius JW, Thron T, Janssen S, Shastri GG, Ilhan ZE, Challis C, Schretter CE et al (2016) Gut microbiota regulate motor deficits and neuroinflammation in a model of Parkinson’s disease. Cell 167(6):1469-1480.e12. 10.1016/j.cell.2016.11.018 27912057 10.1016/j.cell.2016.11.018 PMC5718049

[55] Aho V, Houser MC, Pereira P, Chang J, Rudi K, Paulin L, Hertzberg V, Auvinen P et al (2021) Relationships of gut microbiota, short-chain fatty acids, inflammation, and the gut barrier in Parkinson’s disease. Mol Neurodegener 16(1):6. 10.1186/s13024-021-00427-6 33557896 10.1186/s13024-021-00427-6 PMC7869249

[56] Chen SJ, Chen CC, Liao HY, Lin YT, Wu YW, Liou JM, Wu MS, Kuo CH et al (2022) Association of fecal and plasma levels of short-chain fatty acids with gut microbiota and clinical severity in patients with Parkinson disease. Neurology 98(8):e848–e858. 10.1212/WNL.0000000000013225 34996879 10.1212/WNL.0000000000013225 PMC8883514

[57] Wang N, Feng BN, Hu B, Cheng YL, Guo YH, Qian H (2022) Neuroprotection of chicoric acid in a mouse model of Parkinson’s disease involves gut microbiota and TLR4 signaling pathway. Food Funct 13(4):2019–2032. 10.1039/d1fo02216d 35103734 10.1039/d1fo02216d

[58] Ostendorf F, Metzdorf J, Gold R, Haghikia A, Tönges L (2020) Propionic acid and fasudil as treatment against rotenone toxicity in an in vitro model of Parkinson’s disease. Molecules 25(11):2502. 10.3390/molecules25112502 32481507 10.3390/molecules25112502 PMC7321113

